# Evolution of Cooperation: Combining Kin Selection and Reciprocal Altruism into Matrix Games with Social Dilemmas

**DOI:** 10.1371/journal.pone.0063761

**Published:** 2013-05-22

**Authors:** Som B. Ale, Joel S. Brown, Amy T. Sullivan

**Affiliations:** Department of Biological Sciences, University of Illinois at Chicago, Chicago, Illinois, United States of America; Hungarian Academy of Sciences, Hungary

## Abstract

Darwinian selection should preclude cooperation from evolving; yet cooperation is widespread among organisms. We show how kin selection and reciprocal altruism can promote cooperation in diverse 2×2 matrix games (prisoner’s dilemma, snowdrift, and hawk-dove). We visualize kin selection as non-random interactions with like-strategies interacting more than by chance. Reciprocal altruism emerges from iterated games where players have some likelihood of knowing the identity of other players. This perspective allows us to combine kin selection and reciprocal altruism into a general matrix game model. Both mechanisms operating together should influence the evolution of cooperation. In the absence of kin selection, reciprocal altruism may be an evolutionarily stable strategy but is unable to invade a population of non-co-operators. Similarly, it may take a high degree of relatedness to permit cooperation to supplant non-cooperation. Together, a little bit of reciprocal altruism can, however, greatly reduce the threshold at which kin selection promotes cooperation, and vice-versa. To properly frame applications and tests of cooperation, empiricists should consider kin selection and reciprocal altruism together rather than as alternatives, and they should be applied to a broader class of social dilemmas than just the prisoner’s dilemma.

## Introduction

Darwinian selection precludes any action that increases the competitive ability of others at the expense of one’s self [1]. Yet in social species, kinship fosters cooperation and common agendas help unrelated individuals to work together within and between species. Cooperation abounds in nature on many levels of biological organizations [2]–[4], from social insects and social vertebrates [5]–[7] to coevolved pairs of plants and their pollinators [8], symbiosis [9], and even cancer cells [10].

The three standard motives for the evolution of cooperation include kin selection, reciprocity, and group selection [11]–[15]. Hamilton [16] formalized the idea of kinship in promoting the evolution of altruism. Supposing a cost to the altruist (*c*), a benefit to the recipient (*b*), and their genetic relatedness (*r*), Hamilton [16] showed that natural selection favours altruism among relatives when *r>c/b*. The concept is based on ‘inclusive fitness’: the sum of an individual’s reproductive success plus the effects the individual has on the reproductive success of its relatives. For example, sterile worker ants can accrue reproductive benefits by helping their relative, the queen. In so doing, their shared genes survive and get passed on to the next generation.

Parallel to kin selection, Trivers [17] used reciprocity to explain cooperation among non-relatives. Selection may favour altruism toward a non-relative if the recipient later returns the favour, in which case both individuals accrue a net benefit. Because reciprocal altruism is vulnerable to individuals who cheat by failing to repay acts of altruism, Axelrod and Hamilton [18] used an iterated prisoner’s dilemma game to show how a tit-for-tat strategy might prevail against completely non-cooperative strategies.

Kin selection and reciprocal altruism are often viewed and tested as alternatives. Furthermore, theories of kin selection rely on population genetic models while models for reciprocal altruism focus on the specifics of repeated interactions and the capacity to shape conditional strategies. The two have never melded easily [12]–[14].

The germs of kin selection and reciprocal altruism can be traced back to Darwin [1], [19] who also saw how group selection might favour altruism, that is, a group containing many altruists – each ready to subordinate their own selfish interests for the greater good of the group – may have a survival advantage over a group composed mainly or exclusively of selfish organisms. The work of Williams [20] and Maynard Smith [21] ended the ‘good of the species’ tradition in the 1960s. While we are aware of the recent debate over the significance of the theories of kin selection vs. group selection [12]–[14], this is outside this paper’s scope.

Here we formally combine kin selection and reciprocal altruism and show how they are mutually inclusive properties of games. We apply a game theoretic approach [15] to three famous evolutionary games: prisoner’s dilemma, snowdrift, and hawk-dove. Specifically, we 1) examine separately the effect of adding non-random interactions (*r*, the probability that like-individuals interact with like) and repeated interactions (*w*, the probability that one knows one’s opponent); and 2) develop a general model for the evolution of cooperation that satisfies the requirements of all three games. While simple and straightforward, this combined treatment of kin selection and reciprocal altruism across a variety of social dilemmas is necessary for empiricists to properly frame applications and tests of cooperative behaviours.

## Methods

We describe three games – prisoner’s dilemma, snowdrift, and hawk-dove game – separately, to illustrate why individuals should act selfishly and under what conditions they should cooperate. We incorporate elements of both kin selection and reciprocal altruism, and combine all three games into a single general model.

### Prisoner’s Dilemma

The prisoner’s dilemma provides the archetype for games pitting community interests against those of the individual. Two prisoners being questioned each have the choice to either defend the other’s innocence or betray their guilt. It is better for both players to defend rather than betray each other. The highest payoff of all, however, accrues to one who betrays while being defended by the other. The worst outcome accrues to a player that defends a betraying opponent.

As a 2×2 matrix game, the prisoner’s dilemma (Table 1) has strategies of C (cooperate, or defend) and D (defect, or betray). A co-operator bestows a benefit (*b*) to its opponent at cost (*c*) to itself. A defector incurs no costs and bestows no benefits. We assume that *b>c* insuring that two co-operators enjoy a higher payoff than two defectors. While cooperation maximizes group payoffs, a defector will always enjoy higher payoffs than a co-operator when played against the same opponent. For this game, D is the non-invadable evolutionarily stable strategy (ESS), also known as the Nash equilibrium in conventional games [22]. The ESS represents a “no regret” strategy where no one individual can benefit from unilaterally changing its strategy [15].

**Table 1:** The cost-benefit form of the prisoner’s dilemma.

	C	D
C	b−c	−c
D	b	0

Cooperate strategy (C) bestows benefit *b* on its partner and incurs cost *c* to itself, while the defect strategy (D) bestows no benefit and incurs no cost. The matrix entries represent the payoff to an individual using a row strategy when partnered with an individual using a column strategy (here, *b>c >*0).

Defection is an ESS so long as interactions occur at random with respect to strategy, and if the game is played but once. We relax these assumptions by considering non-random interactions and an iterative game by which individuals can gain information about others’ predispositions.

We let *r* be the probability of non-random interactions where like-individuals interact with like, independent of the frequencies of co-operators and defectors in the population [23]. Following many interactions, a portion *r* occurs with like-individuals while the portion 1-*r* occurs with randomly selected individuals (Table 2).

**Table 2:** A modification for prisoner’s dilemma for non-random interactions.

	C	D
C	r(b−c)+(1−r)(b−c)	r(b−c) − (1−r)c
D	(1−r)b	0

The term *r* is the probability of like interacting with like and (1−*r*) the probability of random interactions. Payoffs are to the individual playing the row strategy against an opponent playing the column strategy.

For reciprocal altruism, we let *w* be the probability that an individual knows the strategy of its opponent based on prior knowledge either through direct experience or via observations as part of an iterative game [24]–[26]. This produces yet another modification to the payoff matrix (Table 3). Tit-for-tat (TFT) [18] can be viewed as a strategy that cooperates with strangers and individuals known to cooperate, and withholds cooperation from individuals known to be defectors or cheaters.

**Table 3:** A modification for prisoner’s dilemma for iterative interactions.

	TFT	D
TFT	r(b−c)+(1−r)(b−c)	− (1−w)c
D	(1−w)b	0

The term *w* is the probability of a player knowing the strategy of its partner. The tit-for-tat (TFT) strategy cooperates with strangers or with known TFT individuals. It plays defect with known D individuals.

We combine the matrix models for kin selection (*r>c/b*) and reciprocal altruism (*w>c/b*) into a single model by simultaneously considering non-random interactions (represented by *r*) in an iterative game (represented by *w*). These adjustments to the prisoner’s dilemma yield a new payoff matrix (Table 4).

**Table 4:** Payoff matrix for an iterative prisoner’s dilemma with non-random interactions.

	All-C	TFT	All-D
All-C	r(b−c)+(1−r)(b−c)	r(b−c)+(1−r)(b−c)	r(b−c)+(1−r)( −c)
TFT	r(b−c)+(1−r)(b−c)	r(b−c)+(1−r)(b−c)	r(b−c)+(1−r)(1−w)( −c)
All-D	r(0)+(1−r)(b)	r(0)+(1−r)(1−w)(b)	0

Following many interactions, a portion *r* occurs with a like-individual, and only the portion 1-*r* occurs with the randomly selected individual. All-C interacts with another All-C, both *r* and 1-*r* of the time the interaction generates a payoff of *r*(*b*−*c*)+(1−*r*)(*b*−*c*), with a total payoff of “*r*(*b*−*c*)+(1−*r*)(*b*−*c*)” for All-C and TFT pair of strategies. Likewise, All-C and All-D obtains *r*(*b*−*c*)+(1−*r*)( −c), and so forth.

### Snowdrift

Imagine two drivers who are trapped on opposite sides of a snowdrift. Each has the option of staying in the car or shovelling snow to clear a path. Like the prisoner’s dilemma, it is always best if your opponent cooperates while you do not. Yet, in snowdrift the individual benefits by digging regardless of what the partner does. The benefit is shared whether one or both individuals dig, while the cost is shared only if both dig (Table 5; the payoff matrix in Table 6).

**Table 5:** Payoff matrix for the snowdrift game.

	C	D
C	b/2−c/2	b/2−c
D	b/2	0

Digging out of the snowdrift gives each player a benefit of *b/2*. The cost is born by the digger (C) who splits the cost when both dig, or bears the entire cost as the sole digger. When *b/*2−*c*>0, the ESS is a mixture of C and D individuals. When *b/*2−*c*/2>0>*b*/2−*c*, All-D is the sole ESS, even though there is still a social dilemma where All-C yields higher payoffs than All-D.

**Table 6:** Payoff matrix for an iterative snowdrift with non-random interactions.

	All-C	TFT	All-D
All-C	r(b/2−c/2)+(1−r)(b/2−c/2)	r(b/2−c/2)+(1−r)(b/2−c/2)	r(b/2−c/2)+(1−r)(b/2−c)
			
TFT	r(b/2−c/2)+(1−r)(b/2−c/2)	r(b/2−c/2)+(1−r)(b/2−c/2)	r(b/2−c/2)+(1−r)(1−w)(b/2−c)
			
All-D	r(0)+(1−r)(b/2)	r(0)+(1−r)(1−w)(b/2)	0

### Hawk-dove Game

In the hawk-dove (or chicken) game, opponents have the opportunity to play Hawk and fight (i.e., defect), or play Dove and give way (i.e., cooperate). Payoffs are maximized when both players give way and play Dove (cooperate). Unfortunately, in a world of doves, it pays to defect and play Hawk (defect). This game’s payoffs can be couched in terms of costs and benefits and modified to consider the joint consequences of kin selection and reciprocal altruism (Table 7; the payoff matrix in Table 8).

**Table 7:** Payoff matrix for the hawk-dove game.

	C	D
C	b/2	0
D	b	b/2−c

In hawk-dove game, if both players cooperate, then they split the benefit *b*/2. When one player cooperates and the other defects, the co-operator obtains nothing but the defector the benefit, *b*. If both players defect, they split the benefit, but also incur a cost (*b*/2−*c*).

**Table 8:** Payoff matrix for an iterative hawk-dove game with non-random interactions.

	All-C	TFT	All-D
All-C	r(b/2)+(1−r)(b/2)	r(b/2)+(1−r)(b/2)	r(b/2)+(1−r)(0)
TFT	r(b/2)+(1−r)(b/2)	r(b/2)+(1−r)(b/2)	r(b/2)+(1−r)(w)(b/2−c)+(1−r)(1−w)(0)
All-D	r(b/2−c)+(1−r)(b)	r(b/2−c)+(1−r)w(b/2−c)+(1−r)(1−w)b	r(b/2−c)+(1−r)(b/2−c)

### General Model

Any game with a social dilemma can be considered in terms of kin selection and reciprocal altruism, with the following elements: 1) Games with social dilemmas must be non-zero sum; 2) The strategy of cooperation (C) played against cooperation must produce a higher payoff than the strategy of defect (D) played against D (*a>d* in Table 9); 3) The strategy C played against C must yield a greater payoff than the average payoffs to C and D when C plays against D: a>(*b+c*)/2; and 4) C played against C yields less payoff than D played against C (*c>a*). In a social dilemma, the always-cooperate (All-C) strategy, that is, individuals cooperating in all situations, provides the best collective outcome, but there is an incentive to cheat and play the strategy of defect (D) in a world of co-operators. Always-cooperate strategy is not an ESS. These games have a mixed strategy ESS when *b>d*, and always-defect strategy (All-D) is the global ESS when *d>b*.

**Table 9:** A general payoff matrix for games with social dilemmas.

	C	D
C	a	b
D	c	d

Three conditions hold for social dilemmas: 1) *a>d*, 2) a>(*b+c*)/2, and 3) *c>a*. A mixed strategy is the ESS when *b>d*, and All-D is a global ESS when *d>b*.

We make this a model of kin selection by permitting non-random interactions (*r* >0) with no prior knowledge of one’s opponent (*w* = 0) (Table 10, with its payoff matrix in Table 11).

**Table 10:** A general payoff matrix with *r* as the probability of non-random interactions where like interacts with like.

	C	D
C	r(a)+(1−r)a	r(a)+(1−r)b
D	r(d)+(1−r)c	r(d)+(1−r)d

**Table 11:** A general payoff matrix for reciprocal altruism where *w* is the probability that an individual knows its opponent’s strategy.

	TFT	D
TFT	w(a)+(1−w)a	w(d)+(1−w)b
D	w (d)+(1−w)c	w(d)+(1−w)d

We combine kin selection and reciprocal altruism into a single model, by simultaneously considering non-random and repeated interactions (Table 12).

**Table 12:** Payoff matrix for an iterative general game with non-random interactions.

	All-C	TFT	All-D
All-C	r(a)+(1−r)a	r(a)+(1−r)a	r(a)+(1−r)b
TFT	r(a)+(1−r)a	r(a)+(1−r)a	r(a)+(1−r)(1−w)b+ wd(1−r)
All-D	r(d)+ c(1−r)(1−w)+wd (1−r)	r(d)+(1−r)w(d)+(1−r) (1−w)(c)	r(d)+(1−r)d

## Results

### Prisoner’s Dilemma

In the prisoner’s dilemma game, after many rounds of interactions, a portion *r* occurs with like-individuals while the portion 1-*r* occurs with randomly selected individuals, with the following outcomes (Table 2): Always-cooperate (All-C) strategy is the ESS only when C played against C yields a higher payoff than D played against C,

*r*(*b−c*)+(1*−r*)(*b−c*)>*r*(0)+(1*−r*)(*b*)
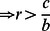
(1)


Always-defect (All-D) strategy is the ESS when the condition is reversed (*r*<*c/b*).

Equation (1) represents Hamilton’s rule (kin selection). It does not rely explicitly on genetic relatedness. The term *r* simply represents the likelihood of assortative interactions. Interactions among relatives generate a positive *r* but relatedness is not necessary. From a game-theoretic modelling approach [23]–[25], kin selection, in a broader sense, represents a form of cooperative behaviour that can evolve when individuals interact non-randomly with respect to strategy.

In reciprocal altruism (when letting *w* be the probability that an individual knows the strategy of its opponent based on prior knowledge) we obtain a different payoff matrix (Table 3).

All-TFT (tit-for-tat strategy) is the ESS when:

*w*(*b−c*)+(1*−w*)(*b−c*)>*w*(0)+(1*−w*)(*b*)
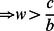
(2)


Note that the always-defect strategy is an ESS for this matrix since 0> *−* (1*−w*)*c*.

Reciprocal altruism only leads to the evolution of cooperation if the probability of recognizing others, *w*, exceeds the cost-to-benefit ratio [24]. Even when tit-for-tat (All-TFT) is an ESS, the strategy of defect continues to be an ESS, although just a local ESS – each can resist invasion from the other, but neither can invade a population of the other. If the population begins with a sufficiently high frequency of defectors, the tit-for-tat strategy cannot invade.

The matrix models for kin selection (*r>c/b*) and reciprocal altruism (*w>c/b*) combine to yield a new payoff matrix when we simultaneously permit non-random interactions (represented by *r*) and an iterative game (represented by *w*) (Table 4, see also [25]).

We can examine when always-cooperate, tit-for-tat, and always-defect strategies are possible ESSs (Table 4):

1. All-C is an ESS when All-C played against All-C yields higher rewards than All-D played against All-C:

*r*(*b−c*)+(1*−r*)(*b−c*)>*r*(0)+(1*−r*)(*b*)
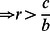

1. TFT is an ESS so long as TFT played against TFT serves better than All-D played against TFT (TFT individuals only defect when opponents defect):

*r*(*b−c*)+(1*−r*)(*b−c*)>*r*(0)+(1*−r*)(1*−w*)(*b*)
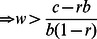
(3)


It is easier for tit-for-tat strategy to be an ESS than it is for the always-cooperate strategy. Individuals using tit-for-tat strategy can benefit both from non-random interactions, and from having an iterative game that provides knowledge of others. If there are no non-random interactions (*r* = 0), tit-for-tat strategy can still be an ESS so long as the probability of recognizing others is greater than the cost-to-benefit ratio: *w>c*/*b* [24].

1. All-D is an ESS so long as All-D played against All-D serves better than TFT played against All-D:

0> *r*(*b−c*)+(1*−r*)(1*−w*)( *−c*)
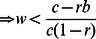
(4)


Decreasing either *w* or *r* increases the likelihood of always-defect being an ESS. When there are no non-random interactions (*r* = 0), defection is always an ESS (even when tit-for-tat strategy is also an ESS).

**Figure 1:**
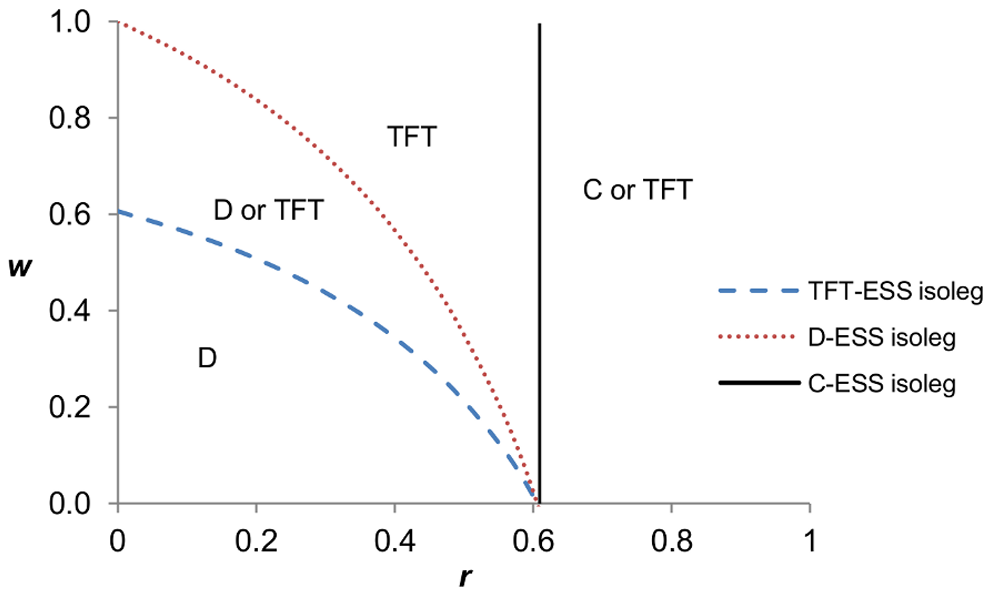
Prisoner’s dilemma with varying combinations of *r* and *w*. Below the D ESS isoleg, All-D is ESS. Above the TFT isoleg, TFT is an ESS. Either D or TFT can be the ESS in the region between the D- and TFT-ESS isoleg, leading to alternative stable states. To right of the C-ESS isoleg, C can be an ESS. Parameter values: *b* = 1.65 and *c* = 1.

We can consider all of the combinations of non-random interactions and foreknowledge of others that permit cooperation to evolve (Figure 1). The three ESS conditions generate three isolegs – lines of indifference in terms of behavioural choice (*sensu* [27]). We shall denote these isolegs as the C-ESS isoleg, TFT-ESS isoleg, and D-ESS isoleg, respectively. They can be found by evaluating the above conditions (equation 4) as equalities rather than inequalities. Each can be solved in terms of *w* as a function of *r*, thus providing the isoleg in the state space of familiarity, *w*, versus non-random interactions, *r*. Outside of the C-ESS (or TFT-ESS) isoleg, always-cooperate (or tit-for-tat) strategy is an ESS (it may be local or global). Inside these isolegs, always-cooperate (or tit-for-tat) is not an ESS. Inside of the D-ESS isoleg, always-defect is an ESS, and outside of this isoleg it is not.

Having both non-random interactions (*r*) and familiarity (*w*) increases the range over which cooperation can evolve, and reduces the likelihood that All-D will be an ESS. Rather than alternative hypotheses for the evolution of cooperation, kin selection and reciprocal altruism can be considered jointly (see also [28], [29]).

The opportunity for cooperative behaviours to evolve via non-random interactions (kin selection) and iterative games (reciprocal altruism with tit-for-tat strategies) extends to other games of social dilemmas, such as the snowdrift and hawk-dove games.

### Snowdrift

The results in snowdrift game are interesting (Table 5) in that always-cooperate (All-C) is never an ESS. When *b*/2– *c* >0, always-defect (All-D) is not an ESS either. Thus, the ESS is a mixture of cooperators (C) and defectors (D) (mixed strategy). When *b-c* >0> *b*/2– *c*, always-defect is an ESS even though always-cooperate would provide each player with higher payoffs. Regardless of whether the snowdrift game has a mixed ESS or an ESS of All-D, it represents a social dilemma.

As in the prisoner’s dilemma, kin selection or reciprocal altruism can allow for All-C to be an ESS. The conditions for cooperation to be the ESS under non-random interactions remains Hamilton’s Rule (*r>c/b*), and for repeated interactions remains *w>c/b*.

We can produce a payoff matrix that simultaneously considers non-random interactions, *r*, and the possibility of knowing one’s partner, *w*, via repeated plays of the game (Table 6).

In snowdrift, the following ESS conditions emerge:

1. All-C is the ESS when *b/*2*−c*/2> *b/2*(1*−r*)

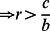

1. TFT is the ESS when *b/*2*−c*/2*> b/*2(1*−r*)(1*−w*)

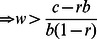

1. All-D is the ESS when 0> *b/*2*+cr*/2*−bw+brw−c+cw−crw*

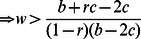



**Figure 2:**
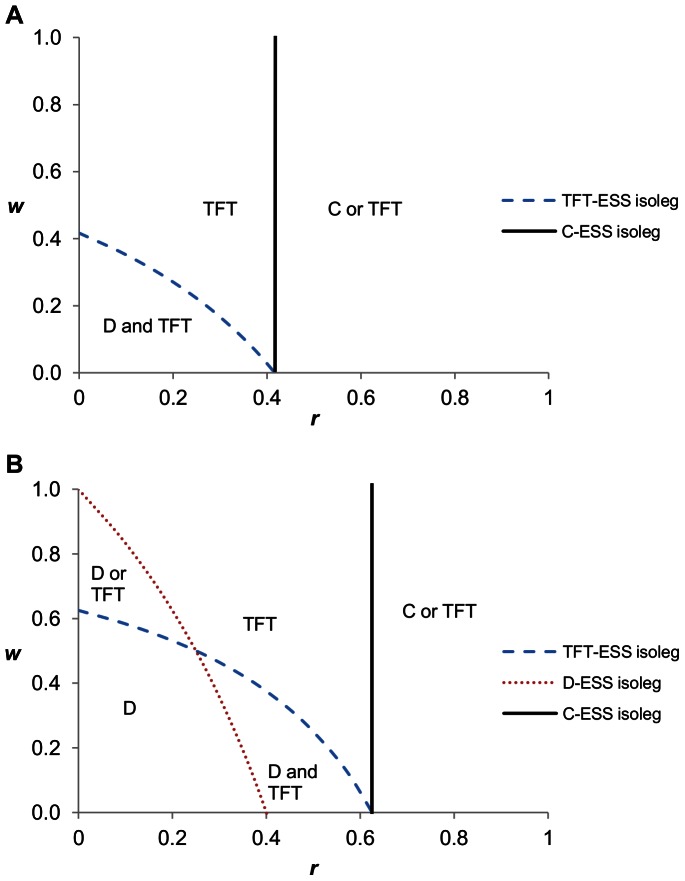
The snowdrift game with varying combinations of r and w, under low and high costs. Panel A shows the snowdrift game under low cost (where *b*/2– *c* >0; values used were *b* = 2.4, *c* = 1). At low *r* and *w*, a mixed strategy of TFT and D is the ESS. At high *r* and *w*, All-TFT becomes the global ESS, and once *r* is sufficiently large, All-C can be an ESS. Panel B shows that higher values of *r* and *w* are required to promote cooperation when costs of cooperating are higher (where *b*/2– *c* <0, values used were *b* = 2.4, *c* = 1.5). Because the D and TFT ESS isolegs cross, there are four regions of outcomes: 1) All-D is a global ESS, 2) All-D or All-TFT is local ESS (alternate stable states), 3) a mixture of TFT and D are a global ESS, and 4) All-TFT is the global ESS. All-C can be an ESS when *r*>*c*/*b*.

We can consider all combinations of non-random interactions and foreknowledge of others that permit cooperation to evolve (Figure 2). For the case of low costs (*b*/2– *c* >0), there are All-C and All-TFT isolegs (Figure 2A). There is no All-D isoleg because All-D is never an ESS for the low-cost snowdrift game. Outside of the All-TFT isoleg, tit-for-tat (TFT) is the global ESS; outside of the All-C isoleg, all-cooperate is an ESS. Inside of the TFT isoleg, a mixed strategy of tit-for-tat individuals and defectors is the ESS. Increasing either *r* or *w* increases the frequency of tit-for-tat individuals within the mixed-strategy ESS and eventually the likelihood that tit-for-tat is the sole ESS.

For high costs (*b*/2– *c* <0), the TFT-ESS isoleg intersects the D-ESS isoleg producing four outcomes for the ESS (Figure 2B). At low levels of non-random interaction and with little likelihood of knowing one’s partner, always-defect is the global ESS. At high levels of knowledge but low levels of non-random interactions, always-defect or tit-for-tat strategy is a local ESS (this region also exists for prisoner’s dilemma). With higher levels of non-random interactions but little knowledge of one’s partner, a mixed strategy of tit-for-tat individuals and defectors comprises a global ESS. It takes high values of both *r* and *w* for tit-for-tat to be the sole global ESS.

### Hawk-dove Game

Like snowdrift, the hawk-dove game has a low and high cost form. Regardless of cost, always-cooperate is never an ESS. With low costs (*b*/2*−c*>0), always-defect is the global ESS – there is no cooperation. With high costs (*b*/2−*c*>0), a mixed strategy of cooperation and defect is the global ESS. High costs and a mixed-strategy ESS are the typical form of the hawk-dove game. Both kin selection and reciprocal altruism permit additional outcomes to the game favouring the evolution of cooperation.

Under strict kin selection (*w* = 0), always-cooperate can be the ESS when *r*>*b*/(*b*+2*c*). Under strict reciprocal altruism (*r* = 0), tit-for-tat can be an ESS when *w*>*b*/(*b*+2*c*). When we modify the hawk-dove game to include both non-random interactions and iterative plays of the game, we get the following matrix (Table 8).

Permitting both kin selection and reciprocal altruism allows the following ESSs:

1. All-C is the ESS when *b*/2> *b*−*cr*−*br*/2

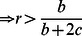

1. TFT is the ESS when *b*/2> *r*(*b*/2−*c*)+(1−*r*)*w*(*b*/2−c)+(1−*r*)(1−*w*)*b*

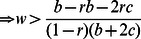

1. All-D is the ESS when *b*/2−*c>r*(*b*/2)+(1−*r*)(*w*)(*b*/2−*c*)

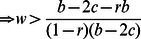



**Figure 3:**
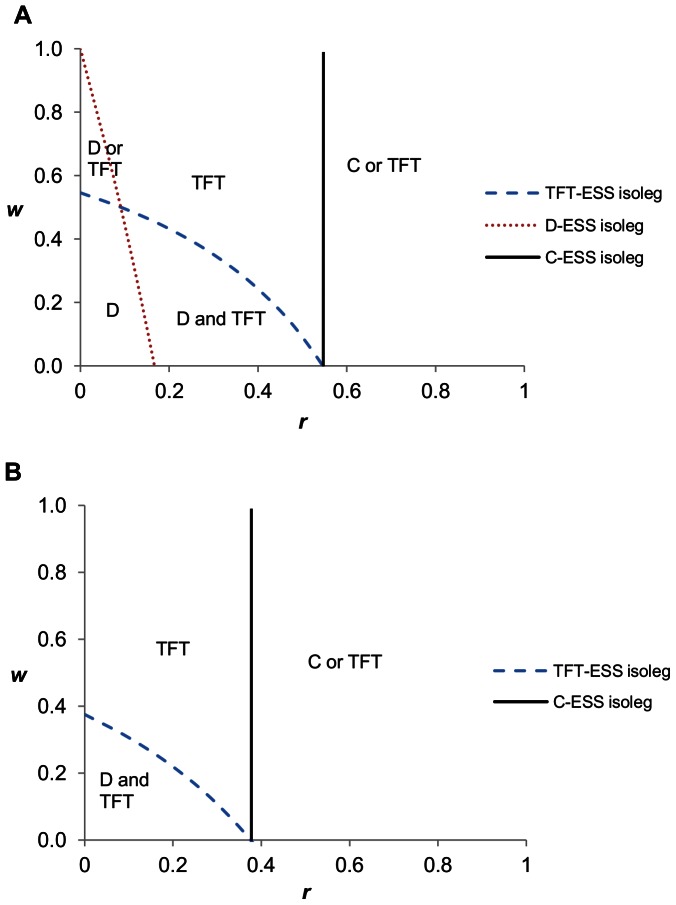
The hawk-dove game at varying values of r and w, under low and high cost. Panel A shows the hawk-dove game under low cost (*b*/2−*c*>0; values used were *b* = 2.4, *c* = 1). The D and TFT isolegs cross, so there are four regions of outcomes: 1) All-D is a global ESS, 2) All-D or All-TFT are local ESSs (alternate stable states), 3) a mixture of TFT and D are a global ESS, and 4) All-TFT is the global ESS. All-C can be an ESS when *r*>*c*/*b*. Panel B shows the hawk-dove game under high cost (*b*/2−*c*<0; values used were *b* = 2.4, *c* = 2) when. At low values of *r* and *w*, a mixed strategy of TFT and D is the ESS. At higher values TFT becomes the global ESS. Once *r* is sufficiently large, then All-C can be an ESS.

The low cost hawk-dove game generates the same configuration of ESSs as the high cost snowdrift game (Figure 3A). Always-defect is the global ESS at low *r* and *w*. At higher *w* and low *r*, always-defect or tit-for-tat is the local ESS resulting in alternate stable states. A mixed strategy ESS results at high *r* but low *w*. With alternate stable states, a small increase in non-random interactions can make tit-for-tat the global ESS. Similarly, with a mixed strategy ESS, a small increase in the knowledge of one’s opponent can make tit-for-tat the global ESS.

The high cost hawk-dove game produces the same outcomes as the low cost snowdrift game. There is no D-ESS isoleg. Hence the ESSs can be a mixed strategy solution (low *r* and *w*), tit-for-tat as the global ESS, or always-cooperate as an ESS once the level of non-random interactions is sufficiently high (Figure 3B). A relatively small likelihood of non-random interaction or knowledge of one’s opponent is sufficient to produce a cooperative solution.

### General Model

The findings from our general model (Table 9) – where cooperatives playing against cooperatives produce a higher payoff than defectors playing against defectors – reveal a mixed strategy ESS.

For All-C to be ESS, *r*(*a*)*+*(1−*r*)*a>r*(*d*)+(1−*r*)*c*

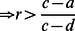



For All-D to be ESS, *r*(*d*)+(1−*r*)*d>r*(*a*)+(1−*r*)*b*

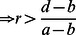



Both All-C and All-D may be local ESSs (alternate stable states) when 
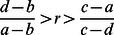
. The ESS is a mixed strategy when: 
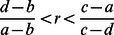
.

Changing this model to reciprocal altruism (by allowing iterative plays of the game, when *w* >0 and *r = *0, All-D is always an ESS (Table 11).

For All-TFT to be an ESS, *w*(*a*)*+*(*1*−*w*)*a>w* (*d*)+(1−*w*)*c* 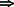 *w*>(*d*−*a)/(c*−*d).*

When we combine kin selection and reciprocal altruism into a single model, by simultaneously considering non-random and repeated interactions (Table 12), we get the following outcomes –.

1 For All-TFT to be ESS:

*a*>*r*(*d*)+(1−*r*)(1−*w*)*c*+(1−*r*)*wd*

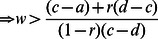



When *r* = 0, then *w*>(*c*−*a*)/(*c*−*d*), and *w* must be positive for any cooperation to evolve.

2 For All-D to be ESS,

*d*>*r*(*a*)+(1−*r*)(1−*w*)*b*+(1−r)(*wd*)
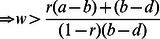



## Discussion

Prisoner’s dilemma, snowdrift, and hawk-dove games pose a dilemma for Pareto efficiency. The natural inclination of an individual player is to defect, even though both players would benefit from cooperation. Our model shows various “doses” of reciprocity and kin selection together can generate cooperation as the sole ESS, both cooperation and defection as local ESSs, cooperation and defection as mixed strategy ESS, and defection as the sole ESS.

Research on kin selection and reciprocal altruism have followed independent paths [4], [12]–[14], [29], [30]–[31]; at times creating controversies and confusing the students of behaviour [13], [32]. Unlike existing models, the model we develop presents kin selection and reciprocal altruism as mutually inclusive properties of games. Both operate through similar processes: the cost-to-benefit ratio of behaving cooperatively must be either less than the probability of like-individuals interacting under non-random interactions (*r*), or the probability of knowing the identity of one’s opponent (*w*). We suggest that non-random and iterative interactions occurring together ease the evolution of cooperation: non-random interactions (kin selection) reduce the threshold of *w* necessary for cooperation, and iterative interactions that increase the probability of knowing one’s opponent reduces the threshold of *r* necessary for cooperation. A similar finding on the importance of joint effect of assortment (kin selection) and reciprocity for understanding cooperation has recently been made [33]. Here we consider three evolutionary games (prisoner’s dilemma, snowdrift and hawk-dove) making it more general. Furthermore, we illustrate the concepts of isolegs [27] to map the boundaries of possible solutions of local, global and mixed strategies.

Modellers’ preoccupation with genetic relatedness (*r*) may have obscured the connections between reciprocal altruism and kin selection. This may obscure the ecological factors promoting assortative interactions and iterated games. For instance, in many vertebrate species, the direct benefits of helping may be sufficient to maintain cooperative societies [6], [7]. A “strategy” rather than a “genetic” approach [15] to cooperation shows how these two processes likely work together – non-random interactions (kin selection) facilitate the evolution of cooperation via reciprocal altruism, and vice-versa.

Although we explicitly integrated kin selection and reciprocal altruism, with matrix model, we note that other mechanisms besides the synergistic conditions generated by kin selection and reciprocal altruism may influence the evolution of cooperation [34]. The introduction of spatial structure via nearest neighbor interactions, for instance, may act to cluster co-operators together [35], [36] promoting cooperation (analogous to non-random interactions, *r*). Such game theoretical models show a transition from models that consider well-mixed populations to spatial grids and complex networks [34]. The extension of these models to coevolutionary strategies, together with the main strategies (cooperate or defect), suggests that the networks themselves “evolve” or “adapt” [37]. In this way the probabilities of assortative interactions and/or familiarity with opponents may change as well and become potential behavioural or evolutionary strategies. Such contributions may help us understand social dilemmas more accurately and push theoretical underpinning to more realistic conditions [38].
